# Supplementary material for: Tumor-induced MDSC act via remote control to inhibit L-selectin-dependent adaptive immunity in lymph nodes
Source: eLife. 2016 Dec 8;5:e17375. doi: 10.7554/eLife.17375 (PMC5199197; doi:10.7554/eLife.17375)
Supplement: Supplementary file 1. — App, application; FC, flow cytometry; IF, immunofluorescence histology; Activ, T cell activation; Mag, magnetic isolation or depletion; Dep; in vivo antibody-mediated depletion. DOI: http://dx.doi.org/10.7554/eLife.17375.026 [file elife-17375-supp1.docx]

| **Target** | **App** | **Source** | **Clone** | **Conjugate** | **Concentration** |
| --- | --- | --- | --- | --- | --- |
| Human CD19 | FC | Biolegend, San Diego, CA | HIB19 | BV421 | 1:20 |
| Human CD3 | FC | BD Biosciences, San Jose, CA | SK7 | PE-Cy7 | 1:20 |
| Human CD4 | FC |  | RPA-T4 | APC-H7 | 1:20 |
| Human CD8 | FC |  | HIT8a | FITC | 1:20 |
| Human CD45RA | FC |  | HI100 | PerCP-Cy5.5 | 1:20 |
| Human CD62L | FC |  | SK11 | BV786 | 1:20 |
| Mouse F4/80 | FC | Biolegend | BM8 | PE | 1:100 |
| Mouse CD3 | FC | BD Biosciences | 17A2 | AlexaFluor 700 | 1:50 |
| Mouse CD4 | FC |  | RM4-5 | FITC | 1:30 |
| Mouse CD4 | FC |  | RM4-5 | V450 | 1:50 |
| Mouse CD8a | FC |  | 53-6.7 | PE-Cy7 | 1:200 |
| Mouse CD11a | FC |  | 2D7 | FITC | 1:50 |
| Mouse CD11a | FC |  | 2D7 | PE | 1:100 |
| Mouse CD11b | FC |  | 1M/70 | PE-Cy7 | 1:300 |
| Mouse CD44 | FC |  | IM7 | FITC | 1:50 |
| Mouse CD44 | FC |  | IM7 | PE | 1:100 |
| Mouse CD45 | FC |  | 30-F11 | PerCP | 1:100 |
| Mouse CD45 | FC |  | 30-F11 | BUV395 | 1:300 |
| Mouse CD45.1 | FC |  | A20 | BUV395 | 1:100 |
| Mouse CD62L | FC |  | Mel-14 | APC | 1:100 |
| Mouse CD62L | FC |  | Mel-14 | PE | 1:200 |
| Mouse B220 | FC |  | RA3-6B2 | PE-CF594 | 1:300 |
| Mouse CCR7 | FC |  | 4B12 | BV421 | 1:50 |
| Mouse Gr-1 | FC |  | RB6-8C5 | FITC | 1:50 |
| Mouse Gr-1 | FC |  | RB6-8C5 | V450 | 1:50 |
| Mouse IFN-γ | FC |  | XMG1.2 | APC | 1:50 |
| Mouse Gr1 | IF | BD Biosciences | RB6-8C5 | FITC | 0.25 μg/ml |
| Mouse B220 | IF |  | RA3-6B2 | PE-CF594 | 1 μg/ml |
| Mouse CD3 | IF | eBioscience, San Diego, CA | 17A2 | eFlour 450 | 5 μg/ml |
| Mouse CCL21 | IF | R&D Systems, Minneapolis, MN | 59106 | N/A | 10 μg/mouse |
| Mouse ICAM-1 | IF | BD Biosciences | 3E2 | N/A | 50 μg/mouse |
| Mouse PNAd | IF |  | MECA-79 | N/A | 20 μg/ml |
| Mouse CD31 | IF |  | MEC 13.3 | N/A | 20 μg/ml |
| Rat IgG | IF | Jackson ImmunoResearch, West Grove, PA | polyclonal | FITC | 1:50 |
| Ar hamster IgG | IF |  | polyclonal | TRITC | 1:50 |
| Rat IgM | IF |  | polyclonal | FITC | 1:50 |
| CD3/CD28 | Activ | ThermoFisher, Waltham, NY | N/A | Magnetic bead | 1 μl/100 μl |
| Mouse Gr-1 | Mag | Miltenyi Biotec, San Diego, CA | M1/79.15 | Magnetic bead | 1:5 |
| Mouse CD4 | Mag |  | L3T4 | Magnetic bead | 1:10 |
| Mouse CD8 | Mag |  | Ly-2 | Magnetic bead | 1:10 |
| CD8^+^ kit | Mag |  | polyclonal | Biotin | 1:5 |
| Mouse Gr-1 | Mag | BD Biosciences | RB6-8C5 | Biotin | 5 μg/ml |
| Mouse Gr-1 | Dep | BioXCell, West Lebanon, NH | RB6-8C5 | N/A | 200 μg/mouse |
| Isotype | Dep |  | LTF-2 | N/A | 200 μg/mouse |

**Supplementary File 1. Supporting information for antibodies used in current study.** App, application; FC, flow cytometry; IF, immunofluorescence histology; Activ, T cell activation; Mag, magnetic isolation or depletion; Dep; *in vivo* antibody-mediated depletion.
